# Eating disorder subtypes differ in their rates of psychosocial improvement over treatment

**DOI:** 10.1186/2050-2974-2-2

**Published:** 2014-01-13

**Authors:** Allison C Kelly, Jacqueline C Carter

**Affiliations:** 1 Department of Psychology, University of Waterloo, Waterloo, ON N2L 3G1, Canada; 2 Department of Psychology, Memorial University of Newfoundland, St. John’s, NL A1B 3X9, Canada

**Keywords:** Anorexia nervosa, Transdiagnostic, Treatment process, Treatment outcome, Change trajectories, Self-compassion, Received social support, Shame, Social safeness

## Abstract

**Background:**

Individuals with Anorexia Nervosa (AN) are renowned for their poor short- and long-term treatment outcomes. To gain more insight into the reasons for these poor outcomes, the present study compared patients with AN-R (restrictive subtype), AN-BP (binge-purge subtype), bulimia nervosa (BN), and eating disorder not otherwise specified (EDNOS) over 12 weeks of specialized eating disorders treatment.

Eighty-nine patients completed the Eating Disorder Examination- Questionnaire (EDE-Q) and various measures of psychosocial functioning at baseline, and again after weeks 3, 6, 9, and 12 of treatment.

**Results:**

Multilevel modeling revealed that, over the 12 weeks, patients with AN-BP and AN-R had slower improvements in global eating disorder pathology, shape concerns, and self-compassion than those with EDNOS and BN. Patients with AN-BP had slower improvements in shame, social safeness (i.e., feelings of warmth in one’s relationships), and received social support compared to those with AN-R, BN, and EDNOS.

**Conclusions:**

These findings support the need for more effective and comprehensive clinical interventions for patients with AN and especially AN-BP. Results also highlight not-yet studied processes that might contribute to the poor outcomes AN patients often face during and after treatment.

## Background

Individuals with Anorexia Nervosa (AN) typically fare worse than those with other eating disorders, and it is unclear why [1-3]. Patients with AN are more likely to drop out of treatment prematurely and less likely to experience sustained symptom remission following treatment [4-9]. They also face a risk of mortality that tends to be higher than that of other psychiatric groups [10-12]. Although it is known that AN is a persistent and destructive illness, we know little about why long-term outcomes are poorer for this eating disorder than for others.

In an attempt to understand the dismal outcomes associated with AN, many researchers have sought to identify prognostic factors within AN-only samples. With regards to pre-treatment predictors, certain characteristics of the disorder, such as binge-purge behaviours and a lower BMI, and various psychosocial variables, such as perfectionism, obsessive-compulsive traits, and socially inhibited interpersonal patterns, have emerged as predictors of poorer outcome over time [13-20]. Certain discharge variables have also predicted long-term outcome in AN. Relapse appears more likely among patients who leave treatment with a greater drive to exercise, residual concerns about weight and shape, and a lower readiness to maintain treatment gains [20,21]. Alcohol abuse and poor social adjustment at discharge have also predicted elevated mortality risk at long-term follow-up [12]. Pre-treatment and post-treatment patient factors therefore seem to be important predictors of long-term outcomes in AN.

### Next research steps

Although the field has identified numerous pre- and post-treatment predictors of outcome among individuals with AN, there are two gaps in the literature which, if addressed, might help to explain why AN patients fare worse than those with other eating disorders. First, there is a scarcity of research on the processes that occur for AN patients *during* treatment [16,22,23]. Most treatment studies assess patients at two discrete time points (i.e., pre and post). Associated analytic approaches typically exclude patients for whom an observation is missing, leaving a biased sample. In addition, linear change is assumed, but never confirmed. We therefore suggest that it is important to assess patients’ functioning at various time points over the course of treatment to allow for analytical approaches that can more precisely model patterns of change. Identifying the ways in which symptoms and psychosocial functioning change – or remain the same – during treatment might shed light on the poor long-term outcomes AN patients face.

Second, there is little research on the ways in which eating disorder diagnostic groups differ in their experiences over the course of eating disorders treatment. This relative absence of research may stem from practical issues that make between-group comparisons difficult during treatment. First, patients with severe AN often attend different treatment programs from those with other eating disorder patients. Second, weight restoration is generally the most studied outcome variable in AN, but is often not a relevant indicator of progress in other eating disorder groups. We suggest value in designing studies that overcome these barriers and render comparisons between diagnostic groups possible. Such studies may shed light on how individuals with AN differ from those with other eating disorders, not only at the start and end of treatment, but also as treatment unfolds.

### The present study

We sought to address the aforementioned gaps in the literature on AN by comparing the change processes of patients with AN-R, AN-BP, BN, and EDNOS during specialized eating disorders treatment. We believed such an approach would help us glean important information about the factors we may be failing to adequately assess and target during treatment. Given the prognostic relevance of eating disorder symptoms and psychosocial factors in predicting long-term outcomes, we sought to compare the trajectories of eating disorder subtypes in both domains. We therefore administered multiple repeated measures of variables that have transdiagnostic relevance to eating disorder patients, rendering between-group comparisons possible. We assessed eating disorder symptoms with the Eating Disorders Examination Questionnaire (EDE-Q) and assessed psychosocial functioning with measures of shame, social safeness, received social support, and self-compassion.

Gilbert’s transdiagnostic theory of psychopathology [24,25] inspired our selection of psychosocial variables. Shame is a painful self-conscious feeling that derives from seeing oneself as defective and imagining others share this view. Gilbert proposed that shame contributes to the maintenance of self-destructive behaviours seen in many forms of psychopathology including eating disorders. Feelings of social safeness, however, are considered antidotal to shame. Social safeness is an emotional sense of warmth, belonging, and calmness, and is thought to arise from the soothing system in the brain, which is sensitive to signals of compassion from others and self [26,27]. According to Gilbert, the soothing system is paramount to sustained well-being because it evolved to tone down the threat system, which is highly implicated in shame and psychopathology [28,29]. Gilbert proposed that receiving compassion from others and/or self is the optimal ways to stimulate the soothing system. Therefore, we additionally examined received social support, as an indicator of compassion received from others, and self-compassion, defined as a kind, caring attitude toward personal distress and shortcomings [30].

We hypothesized that, compared to patients with BN and EDNOS, those with AN would have slower improvements over time in eating disorder pathology, shame, social safeness, received social support, and self-compassion. Given past research documenting the poor prognoses associated with AN-BP, we expected that these slower rates of improvement in AN would be especially pronounced among those with the AN-BP subtype of the illness.

## Methods

### Sample

**Figure 1:**
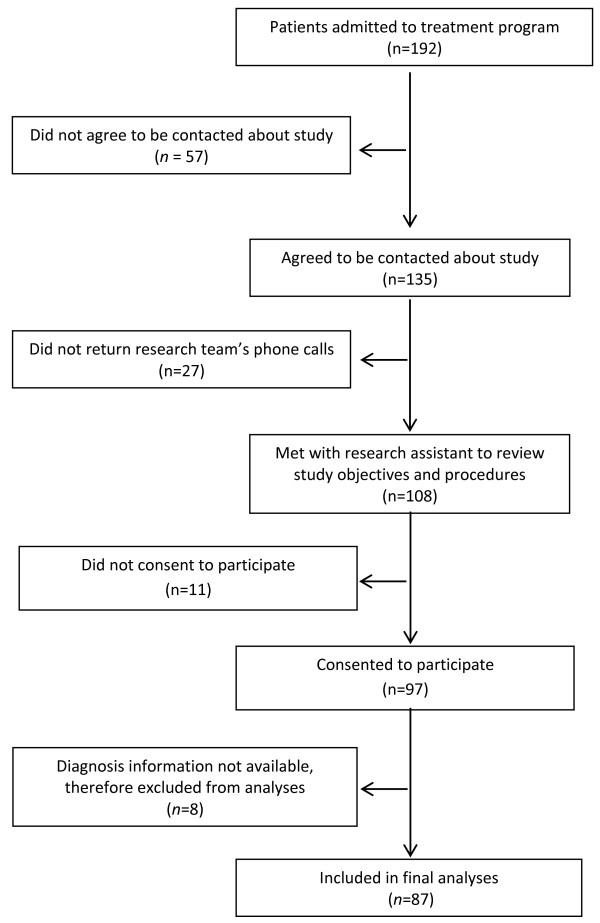
Recruitment and participation flow-chart.

Participants were 89 patients admitted to the Toronto General Hospital’s adult eating disorders program between September 2010 and August 2012. To be admitted into either program, individuals must meet DSM-IV-TR criteria for an eating disorder based on the Eating Disorder Examination [31]. Exclusion criteria for treatment included a diagnosis of binge eating disorder, having an active substance-related disorder, being younger than 18, and being unwilling to comply with the treatment norms. Please see Figure 1 for a flow chart of the recruitment process. The majority (72.2%) of participants was admitted to the eating disorder program’s day hospital, and 27.8% was admitted to the program’s inpatient unit.

Enough information was collected during intake assessments to classify patients based on revised DSM-5 criteria. We therefore present the DSM-5 diagnostic breakdown of our sample, but for simplicity, we group those with specified and unspecified eating disorders under EDNOS. Twenty-six (29.2%) participants had AN-R, 17 (19.1%) had AN-BP, 26 (29.2%) had BN, and 20 (22.5%) had EDNOS. BMI at admission differed by diagnostic group; means were 15.8 (SD = 1.97) for AN-R, 17.08 (SD = 1.54) for AN-BP, 24.52 (SD = 6) for BN, and 23.02 (SD = 4.15) for EDNOS. Of those with EDNOS, three had subthreshold AN-R, four had subthreshold AN-BP, five had subthreshold BN, four had purging disorder, and four had unspecified eating disorders. Eight of those with EDNOS had BMIs between 18 and 20, and the remaining 12 had BMIs of 20 and above. The mean number of previous admissions into the treatment program was 1.29 (SD = .77) and this number did not differ by diagnostic group.

Our sample was predominantly female (97%) and the ethnic breakdown was 79.2% Caucasian, 4.5% East Asian, 1.4% South Asian, 2.8% African-Canadian, 10.8% Latino, and 1.5% mixed race. Participants’ mean age was 28 years (SD = 9.6); a minority of patients (17.4%) was between 17 and 19 years of age and the remainder was 20 and older. The diagnostic groups did not differ in any of our demographic variables.

### Treatment programs

The day hospital and inpatient eating disorders programs at the Toronto General Hospital are run by multidisciplinary teams, consisting of psychiatrists, psychologists, nurses, dieticians, social workers, and occupational therapists. Treatment consists of group therapy as well as staff-supported meals and snacks. The program focuses on medical stabilization, nutritional rehabilitation and normalized eating, eradication of symptomatic behaviours, and in the case of underweight patients, weight restoration [32]. Patients are admitted to these programs on an ongoing basis whenever a spot becomes available. As such, group make-up fluctuates over time. The duration of treatment also varies by patient. Those admitted to the day hospital commit to eight weeks of intensive treatment, and then go onto a less intensive version of treatment. Those admitted to the inpatient unit remain in the program until they have reached a BMI of 20, and then move into the day hospital for eight weeks. Unless patients choose to leave treatment prematurely or are asked to leave because of non-compliance, patients across programs remain in some version of treatment for at least 12 weeks.

### Procedure

Ethics approval was obtained from the University Health Network Research Ethics Board. Participants were asked to complete online questionnaires at the start of their admission, and again after three, six, nine, and 12 weeks of treatment. Questionnaires were emailed to them in the form of a web link, and participants were asked to try and complete them within 48 hours of receiving them. On average, participants completed assessments 4.3 (SD = 1.1) weeks apart from one another.

### Measures

#### Eating disorder symptoms

The 36-item Eating Disorder Examination–Questionnaire (EDE-Q) was used to assess eating disorder symptoms over the previous 28 days [33]. Although questionnaires in this study were administered every 21 days, the fact that the average participant completed surveys late (ever 4.3 weeks) made the assessment windows consistent with the EDE-Q time frame. The EDE-Q generates scores from 0 to 6 on four subscales of Shape Concern, Weight Concern, Eating Concern, and Dietary Restraint. These subscales represent transdiagnostic eating disorder symptoms. A global score of eating disorder pathology is calculated by taking the mean of these four subscale scores. The EDE-Q’s test-retest reliability and internal consistency are strong [34]. In the present sample, Cronbach alphas were .96 for the global score, .89 for Shape Concern, .81 for Weight Concern, .88 for Eating Concern, and .83 for Dietary Restraint. Participants had a mean EDE-Q global score of 4.04 (SD = 1.32) at the time of their admission, confirming their clinically severe eating disorder pathology [35].

#### Shame

Shame was assessed using the 25-item Experience of Shame Scale (ESS) [36]. The ESS generates global shame score, computed by taking the mean of all items, as well as subscale scores representing body shame, character shame, and behaviour shame. A factor analysis of all ESS items in our sample supported a one-factor solution. Sample items from the ESS, rated from 1 (not at all) to 4 (very much) include: “Have you felt ashamed of the sort of person you are?” and “Have you tried to cover up or conceal things you felt ashamed of having done?” The ESS has evidence of high test-retest reliability, good discriminant and construct validity, and high internal consistency, the latter of which was reflected by a Cronbach’s alpha of .95 in our sample. Participants had a mean global shame score of 3.15 (SD = .65) at the time of their admission.

#### Social safeness

We assessed social safeness with the Social Safeness and Pleasure Scale (SSPS) [26]. This 11-item measure uses a 0–4 scale to ask participants to rate the extent to which they experience a sense of warmth, reassurance, and belonging in their social relationships. Sample items include: “I feel easily soothed by those around me,” I feel connected to others,” and “I feel a sense of warmth in my relationships with people.” This measure has been found to have strong internal consistency, which was demonstrated by a Cronbach’s alpha of .94 in our sample. Mean social safeness at baseline was 1.4 (SD = .95).

#### Received social support

We measured received social support (RSS) with the Social Provisions Scale (SPS-short) [37]. The SPS-short is a 6-item measure that can be used to assess perceptions that social support would be available if needed (perceived social support), as well as actual social support received (RSS). We administered the RSS version only. Using a 7-point Likert scale, participants rated the extent to which they received each of six social provisions over the previous three weeks: guidance, assistance, emotional closeness, social integration, reassurance of worth, and opportunity for nurturance. Sample items include: “To what extent did another person(s) come to your assistance,” and “To what extent did another person(s) provide you with a sense of emotional security and well-being.” The scale’s Cronbach’s alpha was .80, demonstrating adequate internal consistency. Participants’ mean baseline score was 4.6 (SD = 1.36).

#### Self-compassion

The 12-item Self-Compassion Scale-Short Form (SCS-SF) was used to assess self-compassion [38]. The SCS-SF asks participants to use a 5-point scale to rate their typical reactions at times of personal distress. Sample items include “I try to be kind and patient towards those aspects of my personality I don’t like,” “I try to see my failings as part of the human condition,” and “When something painful happens I try to take a balanced view of the situation.” This shortened version of the Self-Compassion Scale correlates near perfectly with the full, 26-item version [30]. In the current study, the SCS-SF had a Cronbach’s alpha of .85, demonstrating good internal consistency. Participants’ mean self-compassion score at baseline was 2.04 (SD = .68).

#### Patterns of missing data

At baseline (Time 0), participants completed all questionnaires through a study-specific survey with the exception of the EDE-Q, which was administered as part of the clinical intake process. Time 0 responses on the EDE-Q were available for 80 of the 89 participants; eight patients did not complete their questionnaire, and six patients left treatment before returning their responses. Responses for all other study measures (e.g., shame, self-compassion) at Time 0 were available for all 89 participants. On average, participants completed 3.65 out of 5 assessments over the 12 study weeks, with 58 of the 89 participants completing three or more questionnaires.

#### Analytic strategy

Our primary analytic approach was multilevel modeling with maximum likelihood estimation. This is the recommended statistical approach when modeling participant trajectories over time [39]. Two notable advantages of multilevel modeling in longitudinal studies are its ability to model change trajectories without fixed data collection schedules, and its ability to retain data from participants for whom observations are missing at random (MAR). MAR means that the observations that are missing are unrelated to the unobserved value(s) but may be related to the unobserved value(s) through other variables in the model for which observations are not missing [40]. We found evidence to support the MAR assumption in the present data set.

We tested our hypotheses using PROC MIXED in SAS 9.3. Dependent variables were EDE-Q eating disorder symptoms, shame, social safeness, received social support, and self-compassion across all available time points between weeks 0 and 12 of treatment inclusive. Because participants commonly completed surveys later than they were asked, we used time stamps to enter the precise time point in their treatment course (i.e., 3 weeks, 4.5 weeks, etc.) at which they completed each of their surveys for greater precision [41].

All multilevel models contained a fixed and random effects portion, and an unstructured error covariance. We included a random effect for intercept and time. Fixed effects were time, diagnosis (AN-R, AN-BP, BN, EDNOS), Diagnosis x Time, baseline levels of the outcome variable (covariate), and the former’s interaction with time. Demographic variables (e.g., age, marital status, living circumstances), treatment program (i.e., inpatient vs. day hospital), BMI, and their interactions with time were initially included as fixed effects, but these terms were dropped from final models because none was significant.

Preliminary analyses revealed that baseline EDE-Q global and subscale scores varied by diagnostic group; therefore, we sought to control for shared variance between eating disorder pathology and diagnosis in our models. We therefore included the relevant baseline EDE-Q score and its interaction with time as fixed effects in our models. In all models, a significant effect for Diagnosis x Time would indicate that the dependent variables changed over a time as a function of participants’ eating disorder diagnosis. We then probed significant Diagnosis x Time interactions with simple slope estimates, representing rates of change, for each diagnostic group. Contrasts were then used to compare rates of change across AN-R, AN-BP, EDNOS, and BN. In addition, mean point estimates of the dependent variable were calculated and plotted for each diagnostic group at weeks 0, 3, 6, 9, and 12.

## Results

### Preliminary analyses

First, Pearson zero-order correlations were computed between study variables at baseline to provide information on the distinguishability of our dependent variables from one another (see Table 1). Inter-correlations between EDE-Q subscales indicate that these variables shared anywhere from 46% to 69% of their variance, whereas the EDE-Q subscales shared anywhere from 74% to 83% of their variance with the EDE-Q global scale. No other pair of variables shared more than half of their variance indicating that the psychosocial variables are related to one another, and to eating disorder pathology, but are distinguishable constructs.

**Table 1:** Zero-order correlations between study variables at baseline

	**EDE-Q Global**	**EDE-Q Restraint**	**EDE-Q Weight**	**EDE-Q Shape**	**EDE-Q Eating**	**Shame**	**Safeness**	**Self-Compassion**	**RSS**
EDE-Q global	--	.86^a^***	.91^a^***	.91^a^***	.90 ^a^***	.67^b^***	-.49^c^***	-.45^b^***	-.26^c^*
EDE-Q restraint		--	.68 ^a^***	.69 ^a^***	.71^a^***	.52^b^***	-.40^c^***	-.29^b^*	-.26^c^*
EDE-Q weight			--	.83^a^ ***	.75^a^***	.60^b^***	-.40^c^***	-.43^b^***	-.24^c^*
EDE-Q shape				--	.76^a^***	.62^b^***	-.44^c^***	-.43^b^***	-.21^c^
EDE-Q eating					--	.64^b^***	-.49^c^***	-.47^b^ ***	-.22^c^
Shame						--	-.62 ^c^ ***	-.52^d^***	-.24^c^*
Social safeness							--	.61 ^c^ ***	.63 ^c^***
Self-compassion								--	.34^c^***
RSS									--

^a^*n* = 89; ^b^*n* = 87; ^c^*n* = 76; ^d^*n* = 75.

*Note. EDE-Q*, eating disorders examination questionnaire. *RSS*, received social support.

*p < .05; ***p < .001.

Second, a series of multiple regressions was conducted, using PROC GLM in SAS, to determine whether the four diagnostic groups differed on any of our study variables at baseline. We found that diagnosis predicted baseline EDE-Q global scores, *F* (3, 76) = 2.93, *p* < .05, eating concerns, *F* (3, 76) = 3.48, *p* < .05, weight concerns, *F* (3, 76) = 2.78, *p* < .05, and shape concerns, *F* (3, 76) 4.38, *p* < .01, but not restraint, *F* (3, 76) = 1.0, *n.s*. Contrasts revealed that individuals with AN-R reported lower total and subscale scores than the other groups, *p*’s < .01. Controlling for baseline EDE-Q Global, diagnosis also emerged as a significant predictor of baseline social safeness, *F* (3, 74) = 3.32, *p* < .01. Contrasts revealed that on average, the AN-R and AN-BP groups reported feeling less socially safe at baseline than the EDNOS and BN groups, *F* (1, 76) = 8.61, *p* < .01. There was no effect of diagnosis on baseline received social support, shame, or self-compassion, indicating that patients across the four diagnostic groups did not differ from one another in these variables at the start of the study.

### Does diagnosis predict rate of change in outcome variables?

#### Eating disorder symptoms

**Figure 2:**
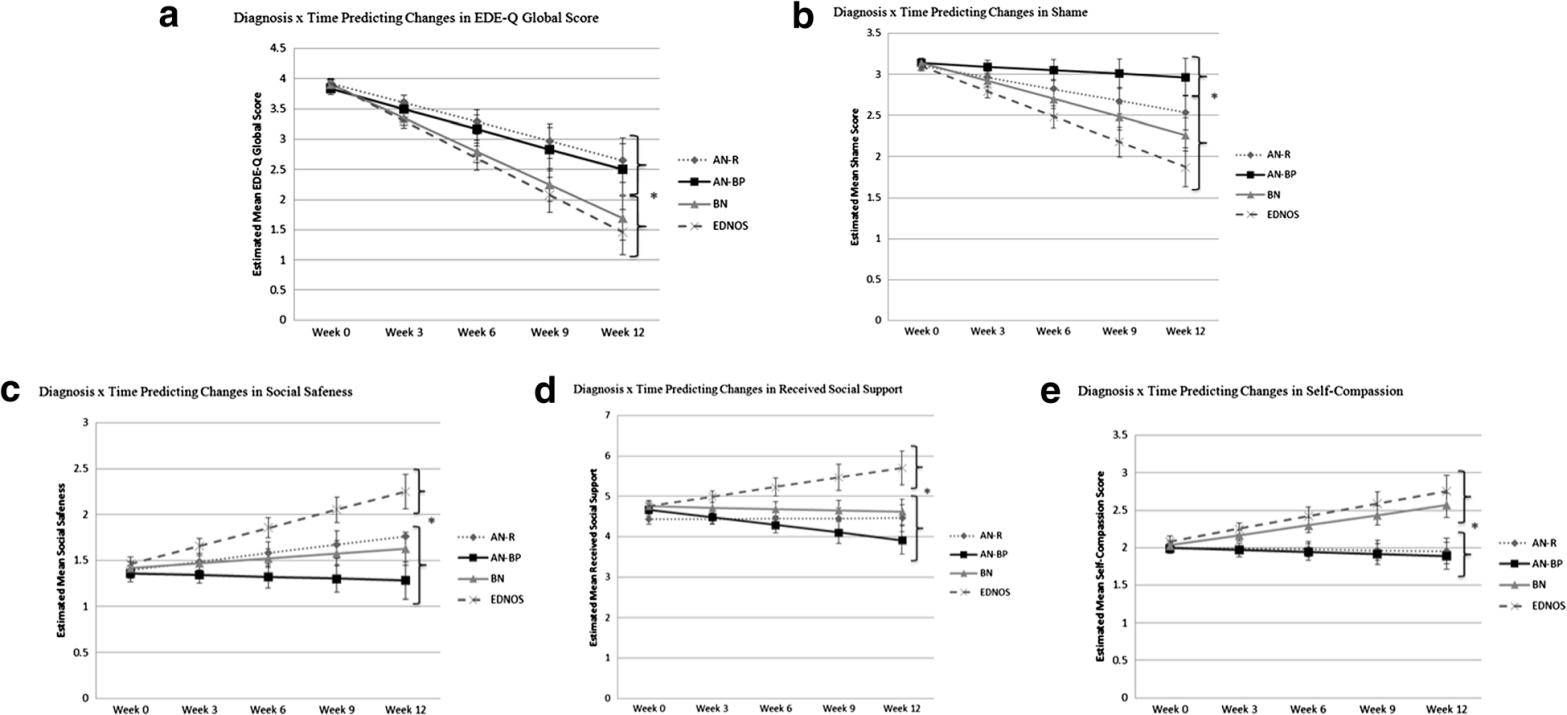
**Diagnosis x time predicting changes in eating disorder symptoms and psychosocial functioning.** Displays estimated means and standard error bars for **a)** EDE-Q global, **b)** shame, **c)** social safeness, **d)** received social support, and **e)** self-compassion at baseline, week 3, week 6, week 9, and week 12 for each diagnostic group.

We first examined EDE-Q Global scores as the dependent variable. A significant effect was found for Diagnosis x Time, *F* (3, 214) = 2.66, *p* < .05. Simple slope estimates revealed that all diagnostic groups had significant decreases in EDE-Q global pathology over time, *p*’s < .001. Simple slope estimates for each diagnostic group are presented in Table 2. Planned contrasts revealed that on average, individuals with AN-R and AN-BP had slower EDE-Q Global decreases over time than those with EDNOS and BN, *F* (1, 214) = 7.81, *p* < .01. Mean point estimates in EDE-Q Global scores were calculated and graphed for each diagnostic group at weeks 0, 3, 6, 9, and 12 (see Figure 2a).

**Table 2:** Estimated rates of change (Slopes) in dependent variables by diagnostic group

	**AN-R**	**AN-BP**	**BN**	**EDNOS**	***Planned Contrasts***
EDE-Q Global	-11 (.03) ***	-.11 (.03) ***	-.18 (.03) ***	-.20 (.03) ***	AN < BN & EDNOS *
EDE-Q Restraint	-.22 (.03) ***	-.16 (.03) ***	-. 21 (.02) ***	-.32 (.03) ***	AN-BP < Others ***
EDE-Q Shape concern	-.00 (.04)	-.04 (.04)	-. 16 (.04) ***	-.15 (.04) ***	AN < BN & EDNOS ***
Shame	-.05 (.02) **	-.01 (.02)	-.07 (.02) ***	-.10 (.02) ***	AN-BP < Others **
Social safeness	.03 (.02) *	-.01 (.02)	.02 (.01)	.07 (.02) *	AN-BP < Others *
					EDNOS > Others *
Received social support	.00 (.04)	-.06 (.03) *	-.01 (.03)	.08 (.03) *	AN-BP < Others *
					EDNOS > Others *
Self-compassion	-.00 (.01)	-.01 (.01)	.05 (.01) ***	.06 (.02) ***	AN < BN & EDNOS ***

*Note. EDE-Q*, eating disorders examination questionnaire. The last column presents the results of planned contrasts between diagnostic groups for dependent variables where Diagnosis x Time emerged as a significant predictor.

*p < .05; **p < .01; ***p < .001.

We then examined each EDE-Q subscale separately as a dependent variable. There was no Diagnosis x Time effect when predicting Eating Concerns, *F* (3, 214) = .74, *n.s*., or Weight Concerns, *F* (3, 214) = 1.38, *n.s*. Diagnosis x Time did, however, predict Restraint, *F* (3, 214) = 5.2, *p* < .01, and Shape Concerns, *F* (3, 214) = 4.3, *p* < .01. With regards to Restraint, simple slope estimates revealed that all diagnostic groups showed significant decreases in dietary restraint over time, *p*’s < .001 (see Table 1). Contrasts additionally revealed that the EDNOS group improved at a faster rate than the average of the other three diagnostic groups, *F* (1, 214) = 12.51, *p* < .001. Furthermore, the AN-BP group improved at a slower rate than the average of the other three groups, *F* (1, 214) = 8.34, *p* < .01. Those with AN-R and BN were similar in their improvements, *F* (1, 214) = .28, *n.s.*

With regards to shape concerns, simple slope estimates revealed non-significant improvements over time for those with AN-R and AN-BP and significant decreases over time for those with BN and EDNOS, *p*’s < .001 (see Table 2). Planned contrasts additionally revealed that those with AN-R and AN-BP improved at a significantly slower rate than those with EDNOS and BN, *F* (1, 214) = 12.12, *p* < .001.

#### Shame

There was a significant effect of Diagnosis x Time predicting changes in shame, *F* (3, 185) = 4.03, *p* < .01. As presented in Table 2, simple slope estimates revealed significant decreases in shame over time in the AN-R, BN, and EDNOS groups. The AN-BP group, however, did not improve over time, and this trajectory differed significantly from the average rate of change of the other three diagnostic groups, *F* (1, 185) = 8.45, *p* < .01 (see Figure 2b).

#### Social safeness

A significant effect was found for Diagnosis x Time, *F* (3, 181) = 2.63, *p* = .05 predicting changes in social safeness. As seen in Table 2, simple slope estimates indicated that the AN-R and EDNOS groups had significant increases in social safeness over time; the AN-BP and BN groups, however, reported no changes in social safeness over time. Planned contrasts revealed that the trajectory of the AN-BP group had a significantly slower change trajectory from the average of the other three diagnostic groups, *F* (1,181) = 5.47, *p* < .05. In addition, the EDNOS group had a significantly faster change trajectory than the average of the other three groups, *F* (1,181) = 5.24, *p* < .05 (see Figure 2c).

#### Received social support

We found a significant effect for Diagnosis x Time predicting changes in received social support, *F* (3, 181) = 2.94, *p* < .05. Simple slope estimates, presented in Table 2, revealed that the AN-R and BN groups had no changes in received social support over time. The AN-BP group, however, had significant decreases in in received support over time, and the EDNOS group had significant increases over time. Once again, the AN-BP group differed significantly in its trajectory compared to the average trajectory shown by the AN-R, BN, and EDNOS groups, *F* (1,181) = 6.02, *p* < .05. The EDNOS group also differed significantly, showing a faster rate of change than the average of the other three diagnostic groups, *F* (1,181) = 6.28, *p* < .05. Figure 2d displays a graphic representation.

#### Self-compassion

Diagnosis x Time predicted changes in self-compassion, *F* (3, 183) = 5.71, *p* < .001. Simple slope estimates, seen in Table 2, indicated non-significant improvements over time for the AN-R and AN-BP diagnostic groups. The BN and EDNOS groups, however, showed significant increases in self-compassion over time. The average rate of improvement in the BN and EDNOS groups was significantly greater than the average of the AN-R and AN-BP groups, *F* (1, 183) = 16.45, *p* < .001. See Figure 2e for a graphic representation.

#### Summary

As presented in Table 2, individuals with AN-BP had slower improvements in dietary restraint, shame, social safeness, and received support compared to those with AN-R, EDNOS, and BN. Individuals with AN-BP and AN-R had comparable trajectories in global eating disorder pathology, shape concerns, and self-compassion, and these rates of change were poorer than those experienced by patients with EDNOS and BN.

## Discussion

This was the first study to our knowledge to compare eating disorder diagnostic groups’ trajectories of change over multiple time points during treatment. Of note, changes in all outcome variables occurred in a linear manner. We found that compared to patients with BN and EDNOS, patients with AN, and especially those with AN-BP, had the slowest improvements in eating disorder symptoms and psychosocial functioning over 12 weeks of treatment. Both the AN-BP and AN-R groups had slower improvements in global eating disorder pathology, shape concerns, and self-compassion over time compared to those with BN and EDNOS. The AN-BP group had slower and generally non-significant improvements in dietary restraint, shame, social safeness, and received social support compared to those with AN-R, BN, and EDNOS. These results suggest previously unknown ways in which patients with AN, and particularly AN-BP, fare worse than other eating disorders patients over the course of specialized treatment.

### Eating disorder pathology

Using the EDE-Q as a transdiagnostic indicator of eating disorder pathology, we found that patients with AN-BP had the slowest improvements in dietary restraint over 12 weeks of treatment. In addition, both AN-BP and AN-R patients had the slowest improvements in global eating disorder pathology and shape concerns. The fact that AN patients generally improved more slowly than other eating disorder patients is perhaps not surprising given what is known about the recalcitrant nature of AN. It is noteworthy, though, that although AN patients had slower improvements in shape concerns over treatment, there was no effect of diagnosis on changes in weight concerns or eating concerns over 12 weeks, suggesting that patients with AN-R and AN-BP only differ from those with BN and EDNOS in their rates of improvement on certain aspects of eating disorder pathology. It is nevertheless important to note that the EDE-Q subscales correlated fairly highly with one another, and with the EDE-Q global score, making it important to replicate this finding in future research.

### Shame and social safeness

Over 12 weeks of treatment, individuals with AN-R, BN, and EDNOS experienced significant increases in social safeness and decreases in shame. Individuals with AN-BP, however, did not show significant improvements in these areas, and their trajectories differed from the average trajectory of the other three groups. These findings suggest that compared to their fellow patients, AN-BP sufferers experienced a more persistent sense of isolation and defectiveness over the course of treatment, with no improvement in feelings of inadequacy and faultiness, or in feelings of social connectedness, warmth, and belongingness. The lack of improvement in shame that AN-BP patients tend to show is particularly concerning in light of growing research documenting the maintaining role that shame plays in the eating disorders [42-44]. Findings are also noteworthy given the group-based nature of our treatment programs, where staff and co-patients are encouraged to be supportive of another. Our results suggest there may something unique about individuals with AN-BP that limits the emotional benefits they are able to derive from group-based therapy.

### Received social support

Individuals with AN-BP reported significant decreases in received social support over time, a trajectory that differed significantly from the average of the three other diagnostic groups. EDNOS patients were the only group to show improvements in received support over time, and those with AN-R and BN reported no changes. Because received support was assessed through self-report, it is difficult to know whether the decreases AN-BP patients reported were objective in nature, or whether they primarily reflected patients’ emotional sense of insecurity and isolation. The measure of received support also does not allow us to determine whether patients responded to items based on their experiences within the treatment context, outside of treatment, or both. In future research, it would be interesting to examine whether certain interpersonal patterns characteristic of AN-BP, such as being cold/distant, socially inhibited, and vindictive/self-centered [19], may lead these patients to lose favour among co-patients and therapists as treatment progresses.

### Self-compassion

Patients with AN-R and AN-BP had slower and non-significant changes in self-compassion over 12 weeks compared to patients with BN and EDNOS. This finding suggests that whereas BN and EDNOS patients come to relate to their distress with more kindness and patience over the course of therapy, individuals with AN do not. Rather, they report no improvements in their self-compassion – that is, their tendency to respond to their feelings of inadequacy with self-kindness rather than self-judgment, mindfulness rather than over-identification, and a perspective that suffering is common to humanity rather than isolating. It would be interesting to know whether these persistently low levels of self-compassion contribute to the poorer outcomes AN patients tend to experience during and after treatment for their eating disorder. Indeed, there is growing evidence that deficits in self-compassion are associated with more severe and prolonged eating disorder pathology in eating disorder patients [45].

### Limitations and future research

This study has a number of limitations. First, it was correlational in nature meaning we must be cautious about interpreting the results. Specifically, we cannot conclude that patients’ eating disorder diagnosis played a causal role in determining their change trajectories. Second, the sample was quite heterogeneous in nature, and treatment intensity varied across patients. Although these sample and treatment characteristics were partly inevitable due to the nature of our research question, it would be advisable to replicate the present findings in a treatment setting where patients across diagnostic groups undergo the same intensity and duration of treatment. Indeed the present results should be viewed as preliminary. Third, there were no follow-up assessments of patients beyond the 12-week study. In future research, it will be interesting to examine the ways in which patients trajectories change or remain the same after treatment. Fourth, although our sample size was relatively large, it will be important to replicate our results in even larger transdiagnostic samples of eating disorder patients.

## Conclusions

The present study provides evidence that patients with AN, and especially those with AN-BP, undergo processes during treatment that differ from those with BN and EDNOS. In particular, their symptoms and psychosocial functioning improve at a slower pace, and often – particularly in the case of AN-BP – at a non-significant pace. Previous research has found that within AN-only samples, poorer psychosocial functioning and more severe illness markers predicted worse outcomes. Our findings extend this research by suggesting that it is not just a subset of AN patients who warrant greater clinical attention. Rather, the trajectories of change AN and especially AN-BP patients showed in our study suggest that compared to patients with BN and EDNOS. Our findings suggest that AN patients may require more comprehensive interventions than those with other eating disorders. It might be important to investigate psychotherapeutic approaches whose integration into current treatments would allow clinicians to better target shame, social safeness, social support, and self-compassion.

## Competing interests

The authors declare that they have no competing interests.

## Authors’ contributions

AK took the lead in devising the study, collecting and analyzing data, and writing up results. JC provided input at all stages. Both authors read and approved the final manuscript.
